# Curcumin–Induced Stabilization of Protein–Based Nano-Delivery Vehicles Reduces Disruption of Zwitterionic Giant Unilamellar Vesicles

**DOI:** 10.3390/molecules27061941

**Published:** 2022-03-17

**Authors:** Ogadimma D. Okagu, Raliat O. Abioye, Chibuike C. Udenigwe

**Affiliations:** 1 Department of Chemistry and Biomolecular Sciences, Faculty of Science, University of Ottawa, Ottawa, ON K1N 6N5, Canada; ookag095@uottawa.ca (O.D.O.); rabio069@uottawa.ca (R.O.A.); 2 School of Nutrition Sciences, Faculty of Health Sciences, University of Ottawa, Ottawa, ON K1H 8M5, Canada

**Keywords:** zwitterionic giant unilamellar vesicles, protein, curcumin, protein-chitosan nanocomplexes, biomembrane, succinylation, bio-nano interaction

## Abstract

Curcumin-loaded native and succinylated pea protein nanoparticles, as well as zwitterionic giant unilamellar vesicles were used in this study as model bioactive compound loaded-nanoparticles and biomembranes, respectively, to assess bio-nano interactions. Curcumin-loaded native protein-chitosan and succinylated protein-chitosan complexes, as well as native protein-chitosan and succinylated protein-chitosan hollow, induced leakage of the calcein encapsulated in the giant unilamellar vesicles. The leakage was more pronounced with hollow protein-chitosan complexes. However, curcumin-loaded native protein and curcumin-loaded succinylated protein nanoparticles induced calcein fluorescence quenching. Dynamic light scattering measurements showed that the interaction of curcumin-loaded native protein, curcumin-loaded succinylated protein, native protein-chitosan, and succinylated protein-chitosan complexes with the giant unilamellar vesicles caused a major reduction in the size of the lipid vesicles. Confocal and widefield fluorescence microscopy showed rupturing of the unilamellar vesicles after treatment with native pea protein-chitosan and succinylated pea protein-chitosan complexes. The nature of interaction between the curcumin-loaded protein nanoparticles and the biomembranes, at the bio-nano interface, is influenced by the encapsulated curcumin. Findings from this study showed that, as the protein plays a crucial role in stabilizing the bioactive compound from chemical and photodegradation, the encapsulated nutraceutical stabilizes the protein nanoparticle to reduce its interaction with biomembranes.

## 1. Introduction

Curcumin-loaded native pea protein (CUR/PPI), succinylated pea protein (CUR/SPPI), native pea protein-chitosan (CUR/PPI/CHI) and succinylated pea protein-chitosan (CUR/SPPI/CHI), as well as hollow native pea protein-chitosan (PPI/CHI) and succinylated pea protein-chitosan (SPPI/CHI) [1], and many other bioactive compound-loaded protein nanoparticles [2,3,4,5,6,7,8] have demonstrated promising physicochemical properties, such as high encapsulation efficiency, zeta potential, water-dispersibility, small size, increased surface area-to-volume ratio, improved shelf life, low polydispersity index, stability at high ionic strength, pepsin-resistant properties, enhanced gastric stability, and sustained release of bioactive compounds. However, there are concerns that changes in the structural and physicochemical properties of proteins, during fabrication or conversion to nanoparticles that promote their food application, could also result in unwanted physiological responses. This is plausible because unfavorable nanoparticle-membrane interaction may impact cell viability, cellular uptake efficacy, exocytosis and endocytosis mechanisms, as well as transepithelial and permeation capacity. The potential outcome of this interaction might affect both the structural integrity of the protein nanoparticles and the gastrointestinal cell membrane, and it could cause inflammation, nanoparticles accumulation, decreased protein digestibility, and considerable side effects in the liver, gut microbiota, and muscles [9,10,11,12]. 

Although a few studies have investigated the mechanism of cytotoxicity of bioactive compound-loaded protein nanoparticles [13], possible dose-dependent in vitro toxicity data have been reported [14,15,16]. For instance, curcumin-loaded zein nanoparticles, functionalized with polydopamine in the presence and absence of dodecamer peptide coating, induced cell death (below 20% cell viability) at a nanoparticle concentration above 5 µg/mL. The nanoparticles induced the generation of reactive oxygen species, two-times higher than those of free curcumin, and promoted apoptosis of C6 glioma cells [14]. Curcumin-loaded human serum albumin nanoparticles, coated with HER2 aptamer, induced higher cytotoxicity in SK-BR3 cell line compared to free curcumin, and reduced cell viability to 36% after 72 h [15]. While free curcumin reduced the viability of MCF-7 cells to ~60%, self-assembled curcumin-loaded human serum albumin nanoparticles induced significant cell death at a concentration of 50 µM [16]. Although the studies demonstrated the anticancer activities of curcumin-loaded protein nanoparticles, no information was provided on the effect of the nanoparticles on healthy cells. The mechanism of cytotoxicity can be evaluated through the characterization of the interaction of protein-based nano-delivery systems and biomembranes occurring at the nano-bio interface. This information is essential in the rational design and applicability of nutraceutical-loaded, food-grade encapsulating agents in functional food formulation. The underlying mechanism could depend on the physicochemical properties of the loaded nano-encapsulating agents and their nature of interaction with the gastrointestinal membrane. For instance, the shape and mechanical properties of single-walled carbon nanotubes, functionalized with adsorbed lysozyme, its monomer, amorphous aggregate, and amyloid fibril formed by lysozyme, coupled with the nature of their noncovalent interaction, played an important role in the leakage of liposome content [12]. Lysozyme proteins did not disrupt the lipid membrane under physiological saline conditions except when they rearranged or aligned with the single-walled carbon nanotube to form a one-dimensional shape [12]. Poly-L-lysine and polyamidoamine dendrimers, as well as semihydrophobic nanoparticles of polystyrene of various sizes, shapes, functional group, flexibility, surface hydrophobicity, and charge density have been reported to interact with lipid membranes differentially [17,18,19]. To date, there is no information available for nutraceutical-loaded protein nanoparticles. The physicochemical properties of the engineered protein nanoparticles, with or without the loaded bioactive compounds, which dictate their delivery function and food matrix compatibility [1,3,4], could also determine the nature of their interaction with the gastrointestinal cell membrane during oral administration.

**Scheme 1:**
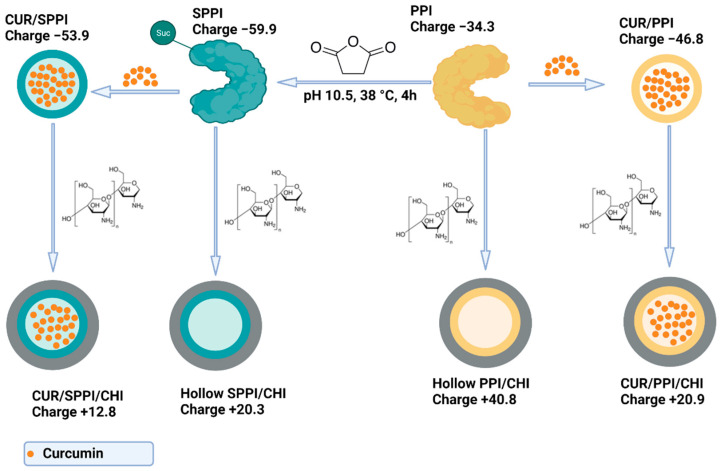
Preparation of hollow and curcumin-loaded protein and protein-chitosan nano-complexes with different physicochemical properties, as previously reported [1].

Our previous study [1] reported the fabrication of hollow and curcumin-loaded pea protein-based nanoparticles (Scheme 1) with the following physicochemical properties: hollow PPI/CHI and SPPI/CHI (size: 151.5 and 176.3 nm, zeta potential: +40.8 and +20.3 mV, respectively, shape: irregular morphology); CUR/PPI and CUR/SPPI (size: 165.6 and 159.9 nm, zeta potential: −46.8 and −53.9 mV, encapsulation efficiency: 34.65% and 24.92%, effective binding constant: 6.9 × 10^4^ M^−1^ and 4.2 × 10^4^ M^−1^, respectively, shape: well-ordered spherical morphology); CUR/PPI/CHI and CUR/SPPI/CHI (size: 194.5 and 183.3 nm, zeta potential: +20.9 and +12.8 mV, encapsulation efficiency: 85.01% and 62.05%, effective binding constant: 14.5 × 10^4^ M^−1^ and 187.9 × 10^4^ M^−1^, respectively, shape: well-ordered spherical morphology). The aim of this study was to assess the implication of the interaction occurring at the bio-nano interface for these well-characterized hollow and nutraceutical-loaded food protein nanoparticles of different physicochemical properties, with giant unilamellar vesicles (GUV) used as model biomembranes. The specific objectives were to evaluate: (1) food-grade nanoparticle-induced leakage or quenching of calcein-loaded giant unilamellar vesicles; (2) the effect of interactions of food-grade nanoparticles on the physicochemical properties of calcein-loaded biomembrane; (3) the impact of the interaction on the structural integrity of the biomembrane.

## 2. Results and Discussion

### 2.1. Nanoparticle-Induced Giant Unilamellar Vesicle Disruption at Physiological pH

**Figure 1:**
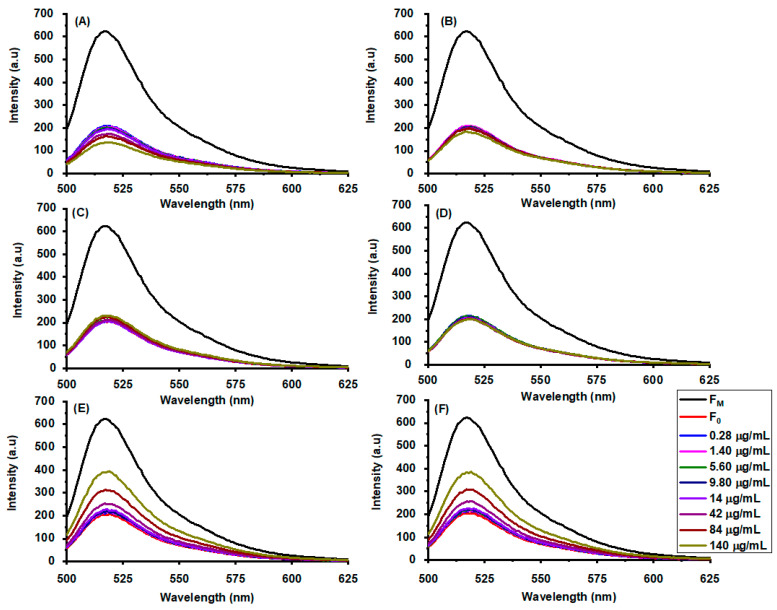
Leakage-induced fluorescence emission spectra of calcein, encapsulated in the GUV, after interaction with various concentrations of (**A**) curcumin-loaded succinylated pea protein, (**B**) curcumin-loaded native pea protein, (**C**) curcumin-loaded succinylated pea protein-chitosan, (**D**) curcumin-loaded native pea protein-chitosan, (**E**) hollow succinylated pea protein-chitosan, and (**F**) hollow native pea protein-chitosan complexes.

**Figure 2:**
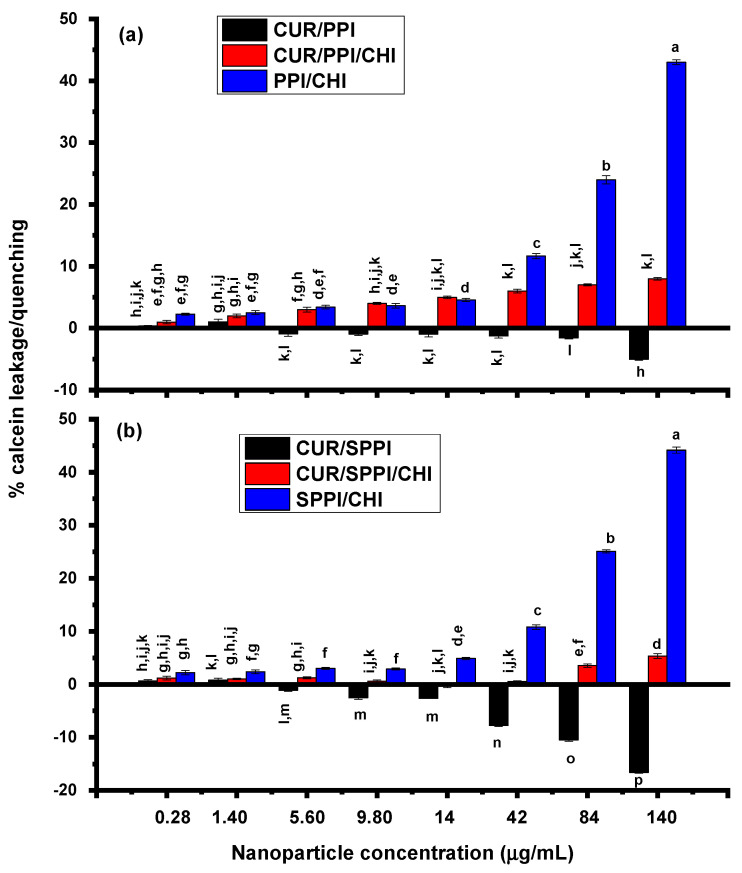
Percentage calcein leakage from the GUV, induced by (**a**) native protein-based nanoparticles and (**b**) succinylated protein-based nanoparticles, with different surface chemistry and physicochemical properties. Bars with similar letters represent mean values that are not significantly different at *p* < 0.05.

There is a lack of studies using leakage assays to investigate the interactions occurring at the bio-nano interface, involving protein-based nanodelivery systems and giant unilamellar vesicles. Existing information on organic compounds, such as antibiotic peptides fengycins [20], maculatin, citropin, and aurein [21], showed the disruption of liposomes as a measure of their antimicrobial properties. Liposome leakage data have also been reported for organic nanoparticles, such as lysozyme functionalized single-walled carbon nanotube, amyloid fibril in nanometer-order and lysozyme aggregates [12], semihydrophobic nanoparticles of polystyrene [19], and nanoscale protein aggregates [22], focusing on cytotoxicity of the nanomaterials. In this work, the leakage-induced fluorescence emission spectra of calcein-loaded giant unilamellar vesicles was monitored, after interactions with various hollow and curcumin-loaded protein nanoparticles of different surface chemistry, size, stability, and biocoating, to study the biophysicochemical interactions that occur at the nano-bio interface. This assay is based on measuring the fluorescence intensity of calcein, which is quenched when encapsulated in the lipid vesicle and fluoresce on leakage. Therefore, the fluorescence intensity is directly proportional to the number of moles of calcein released from the model biomembrane [23]. The fluorescence emission scan (Figure 1) showed that the interaction of the various nanoparticles with calcein-loaded giant unilamellar vesicles did not cause a major shift in the maximum emission wavelength of encapsulated calcein. This indicates that the nanoparticles do not cause any major modification in the structural properties of calcein. The GUV leakage assay (Figure 1 and Figure 2) showed that each nanoparticle pair—CUR/PPI and CUR/SPPI, CUR/PPI/CHI and CUR/SPPI/CHI, or PPI/CHI and SPPI/CHI—demonstrated similar modes of interaction with the GUV. This could be attributed to their similar physicochemical properties, such as chemical composition, size, morphology, and zeta potential [1]. CUR/PPI and CUR/SPPI induced minimal calcein leakage at the lowest nanoparticle concentrations (<5.6 µg/mL). At higher concentrations, a dose-dependent quenching of calcein fluorescence was observed, and the effect was more pronounced with CUR/SPPI than CUR/PPI. The extent of the quenching is quantified by the negative value of percentage leakage (Figure 2). The higher the negative percentage leakage, the higher the quenching effect resulting from the nanoparticle-GUV interaction. Curcumin forms a less stable complex with succinylated pea protein isolate (CUR/SPPI), compared to native pea protein isolate (CUR/PPI), based on their Stern–Volmer binding parameters [1]. The higher stability of CUR/PPI, compared to CUR/SPPI, could have resulted to its lower binding to GUV and, hence, lower fluorescence quenching on interaction. The quenching effect is possibly due to binding of the nanoparticles on the GUV surface without rupturing the membrane. The feasibility of this nanoparticle-membrane association mechanism has been well-established through classic molecular dynamic simulation [24]. The net charge of DOPC GUV is zero, thus ruling out the possibility of electrostatically-induced adsorption. Instead, hydrophobic interaction has been reported to play an essential role in the adsorption of liposomes, with zero net charge, on nanoscale amyloid fibrils derived from lysozymes, and the subsequent disruption of the membrane [22]. In addition, hydrophobic interaction is responsible for the adsorption of carboxyl end-functionalized polystyrene nanoparticles on neutral supported lipid bilayers, dragging the lipids from the model cell membrane to the surface of the nanoparticles and rupturing the membrane at high nanoparticle concentration [19]. 

Interactions of CUR/PPI/CHI or CUR/SPPI/CHI nanoparticles and the GUV induced dose-dependent minimal calcein leakage (Figure 1 and Figure 2), with a more pronounced effect observed in the former. Unlike CUR/PPI and CUR/SPPI, the incorporation of chitosan modified the physicochemical properties of the nanoparticles and resulted in calcein leakage rather than quenching. The binding parameters showed that CUR/SPPI/CHI and CUR/PPI/CHI complexes are significantly more stable than CUR/PPI and CUR/SPPI complexes, and the higher stability resulted in minimal interaction with GUV. The leakage could be attributed to the rupturing of the biomembrane by the nanoparticles. The curcumin-protein and curcumin-protein-chitosan nanoparticles possess opposite surface charges, different sizes, and chemical compositions [1], which could account for their different modes of interaction. The interaction of other nanoparticles of different physicochemical properties, such as amyloid fibril, heat-induced aggregate, and monomer of lysozyme, with liposomes depends on their concentration, shape, and charge. While amyloid fibril induced up to 70% calcein leakage, the lysozyme monomer and aggregates had minimal impact on the liposomes [12]. Carboxylated polystyrene nanoparticles of different sizes, 20 and 100 nm, coated with fetal bovine serum had different effects on the supported lipid bilayer. Unlike the nanoparticle of 20 nm, which had negligible impact on the membrane, quartz crystal microbalance with the dissipation technique showed that 100 nm particles adsorbed on the supported lipid bilayer, which caused a significant decrease in frequency and increase in dissipation to rupture the membrane [25]. Negatively charged carboxyl-functionalized polystyrene nanoparticles induced local gelation in fluid bilayers, whereas positively charged amidine-functionalized polystyrene nanoparticles caused the fluidization of gelled membrane locally [26].

Hollow PPI/CHI and SPPI/CHI nanoparticles showed dose-dependent significant leakage within the tested concentrations. As with the curcumin-loaded protein and protein-chitosan pairs, PPI/CHI and SPPI/CHI nanoparticles displayed similar modes of interaction. There was no significant difference in the leakage effect observed at nanoparticle concentrations of 0.28 to 14 µg/mL. However, major leakage of more than 40% was noted at a nanoparticle concentration of 140 µg/mL (Figure 2). This effect could be attributed to nanoparticle-induced membrane perturbation. Single-walled carbon nanotubes, functionalized with lysozyme from chicken egg white, demonstrated similar dose-dependent leakage of calcein from liposomes, attaining ~30% leakage at a particle concentration of 80 ng/mL. The leakage effect of PPI/CHI and SPPI/CHI hollow could be because of the low stability of the nanoparticles, as demonstrated from their low binding constants (data not shown). The irregular nanostructure of PPI/CHI and SPPI/CHI, coupled with their numerous pores in solution, could also be contributing to the leakage. Pores have been reported to considerably impact membrane integrity [19,27]. Pore-forming toxins employ similar mechanisms to form holes in biomembranes, leading to the death of target cells [28]. Our previous study showed that curcumin encapsulation is essential for the stability of the protein-chitosan complex [1]. The shape of nanoparticles plays an important role in their membrane disruption ability. For instance, one-dimensional shape of single-walled carbon nanotube, coated with lysozyme, was reported to be responsible for liposome disruption [12].

Based on the findings, we propose that the encapsulated guest compound, which stabilizes the host macromolecule used as the delivery vehicle, influences the nature of interaction of hollow or curcumin-loaded protein and protein-chitosan nanoparticles, with biomembranes. Therefore, as the protein or protein-chitosan complexes play a crucial role in stabilizing the bioactive compound from chemical and photodegradation, the encapsulated compound modulates the interaction of the host macromolecule with biomembranes.

### 2.2. Influence of Nanoparticle-GUV Interaction on the Size, Zeta Potential and Polydispersity Index of the Vesicles 

Dynamic light scattering measurements showed that the interaction of CUR/PPI and CUR/SPPI with GUV caused a major reduction in the size of the vesicles. This was unexpected because the calcein leakage assay showed a considerable fluorescence quenching effect above 14 µg/mL, attributed to nanoparticle binding on the GUV surface. Hence, one would expect an increase in the size of the GUV-nanoparticle complex. The reduction in size after binding of the nanoparticles could be because of strong hydrophobic interaction between the nanoparticles and GUV, resulting in the shrinking of the membrane. Another reason could be due to nanoparticle-induced GUV division into smaller particles, while retaining the content of the membrane. The former mechanism seems more likely, as the exposure of PEGylated and non-PEGylated phosphatidylcholine/cholesterol liposomes to serum resulted in the shrinking of the biomembrane [29]. The binding of proteins that are impermeable to the membrane triggered a high osmotic pressure that caused the escape of water from the core of the liposome and, hence, the reduction in liposome size [29]. This mechanism can explain the observed shrinking of GUV after interaction with CUR/PPI and CUR/SPPI, with the membrane selectively allowing the escape of water while retaining calcein at high osmotic pressure, resulting in reduced calcein fluorescence. Other studies have shown that the surface structure and elasticity of supported lipid bilayers, or large unilamellar vesicles, can be reconstructed by nonspecific adsorption of charged nanoparticles [25,26]. For instance, the binding of anionic polystyrene nanoparticles, of different charge density and similar size, on large unilamellar vesicles resulted in the shrinking of the vesicles due to local fluid-to-gel transition of the lipid, which equally caused the reduction in area per molecule and loss of hydration [26]. 

**Figure 3:**
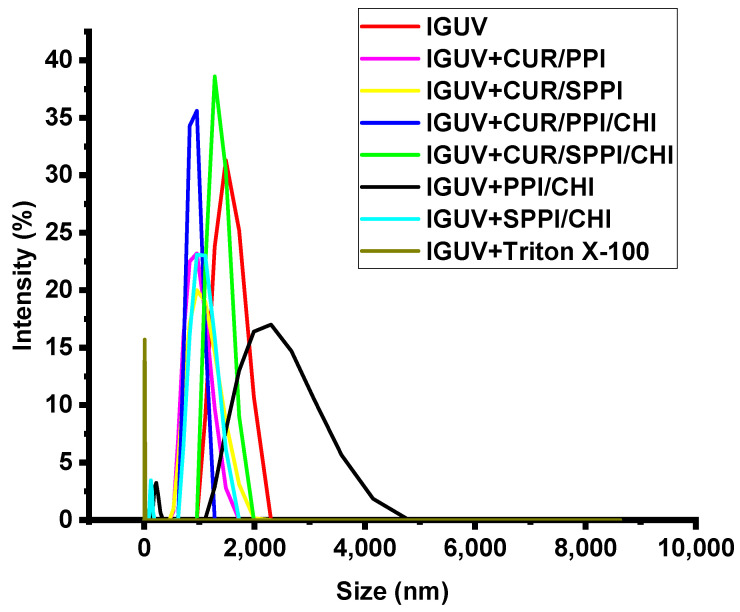
Dynamic light scattering size distribution curve of calcein-loaded giant unilamellar vesicles (lGUV), before and after interaction with hollow (PPI/CHI and SPPI/CHI) and curcumin-loaded protein nanoparticles (CUR/PPI, CUR/SPPI, CUR/PPI/CHI and CUR/SPPI/CHI) and Triton X-100.

**Figure 4:**
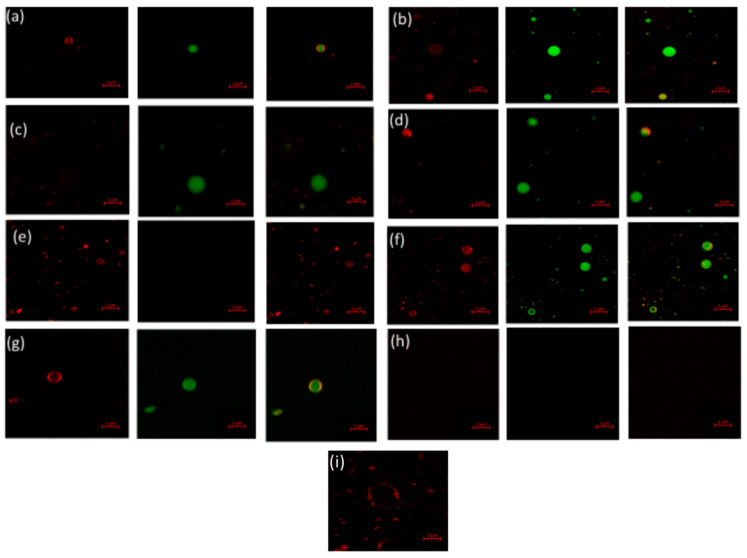
Confocal microscopy images of calcein-loaded GUV, after interaction with (**a**) CUR/SPPI, (**b**) CUR/PPI, (**c**) CUR/SPPI/CHI, (**d**) CUR/PPI/CHI, (**e**) SPPI/CHI, (**f**) PPI/CHI nanoparticles, and (**g**) calcein-loaded GUV, in the absence of nanoparticles, (**h**) calcein-loaded GUV after treatment with Triton X-100 (10%) positive control, and (**i**) GUV without calcein. Images in the first column were acquired at rhodamine B channel (GUV labeled with rhodamine), the second column is at calcein channel, and the third column is the merger of both. Scale bar is 2 µm.

The size of the calcein-loaded GUV, before and after interaction with CUR/PPI/CHI and CUR/SPPI/CHI (Table 1), was comparable, suggesting minimal impact of the nanoparticles on the vesicles. This result supports the minimal GUV leakage (Figure 2) related to the higher stability of the curcumin-protein-chitosan nano-complexes. Although weaker than the effect of 10% Triton X-100, PPI/CHI and SPPI/CHI hollow caused a significant decrease in the size of the GUV (Table 1). This effect could be attributed to direct bursting of the GUV on interaction with the nanoparticles, which corroborates the significant calcein leakage (Figure 2). There was no definite pattern of zeta potential of the calcein-loaded GUV, before and after interaction with the various nanoparticles, as the charges were contributed by the nanoparticles, leakage buffer, or calcein since the zwitterionic GUV has a net zero charge. The polydispersity index of the GUV, after interaction with PPI/CHI and SPPI/CHI hollow (Table 1), indicates the formation of a more heterogeneous mixture (Figure 3). The rupturing of ~40% of the vesicles by the hollow nanoparticles means that the remaining 60% intact GUV resulted in a heterogeneous mixture (Figure 3). The positive control, Triton X-100, decreased the size of the GUV by ~200 folds due to direct bursting and clearing of the vesicles. This is supported by results from confocal (Figure 4h) and widefield fluorescence microscopy (Figure S1i). Triton X-100 is a well-known surfactant that causes almost 100% leakage or disruption of liposome vesicles; thus, it is commonly used to estimate the percentage leakage of fluorophore from loaded vesicles (Section 3.3.1) [20].

### 2.3. Morphological Properties of the GUV after Interaction with Nanoparticles

**Figure 5:**
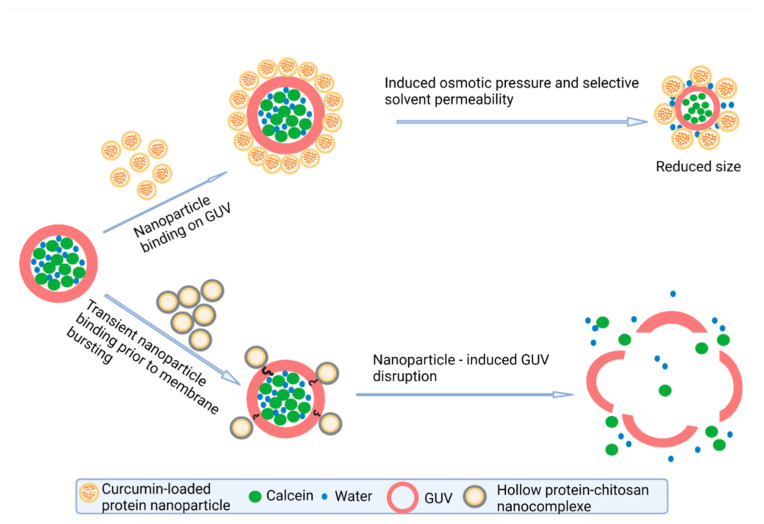
Proposed nanoparticle-giant unilamellar vesicle interaction mechanisms.

The images from confocal and widefield microscopy further corroborate the findings. The morphology of the GUV, after interaction with CUR/SPPI, CUR/PPI, CUR/SPPI/CHI, and CUR/PPI/CHI nano-complexes (Figure 4a–d and Figure S1c–f), looks similar to that of the calcein-loaded GUV in the absence of the nanoparticles (Figure 4g and Figure S1b). This suggests minimal impact of the nanoparticles to the biomembrane. The size range of the nanoparticles < 200 nm [1] falls outside the range of the light microscope, making it difficult to capture the nanoparticles on the surface of the GUV, as postulated for CUR/SPPI and CUR/PPI, or in the vicinity of the GUV for CUR/SPPI/CHI and CUR/PPI/CHI nano-complexes. Figure 4e,f and Supplementary Figure S1g,h confirmed membrane disruption and calcein leakage induced by the hollow SPPI/CHI and PPI/CHI nanocomplexes, with impact more visible in SPPI/CHI. The morphology of the empty GUVs (Figure 4i and Figure S1a) indicates that calcein encapsulation stabilizes the vesicles, thus giving it a definite structure. Taken together, the calcein leakage and microscopy results show two distinct nanoparticle-GUV interaction mechanisms, as illustrated in Figure 5.

## 3. Materials and Methods

### 3.1. Chemical Materials

Chloroform (≥99.5%), 1,2-dioleoyl-sn-glycero-3-phosphocholine (≥99%), 1,2-dioleoyl-sn-glycero-3 phosphoethanolamine-*N*-(lissamine rhodamine B sulfonyl) (ammonium salt) (≥99%), medium molecular weight chitosan, calcein, succinic anhydride (≥99.5%), curcumin (≥94%), and ethanol were purchased from MilliporeSigma Chemical Co., Ltd. (St. Louis, MO, USA). Tris(hydroxymethyl)aminomethane (Tris-base, ≥100.1%), sodium chloride (≥99.0%), Tris-EDTA (99.4–100.6%), and sodium hydroxide (≥98%) were purchased from Thermo Fisher Scientific (Geel, Belgium). Triton X-100 was purchased from VWR (Mississauga, ON, Canada). Yellow pea seed was donated by Pulse Canada (Winnipeg, MB, Canada). Milli-Q water was obtained from a water-purification system (Advantage A10 Q-POD Milli-Q Water System), with total organic carbon level ≤ 5 ppb and resistivity of 18.2 MΩcm at 25 °C. Leakage buffer, which comprises of 1 mM EDTA, 10 mM Tris, and 150 mM NaCl at pH 7.4 was prepared in Milli-Q water. All chemical reagents were of analytical grade and used without further purification.

### 3.2. Synthesis of Hollow and Curcumin-Loaded Protein Nanoparticles

Pea protein isolate (PPI) with total protein content of 96.1%, as determined from Lowry assay, was extracted from yellow pea seeds (*Pisum sativum*) by the isoelectric precipitation technique and succinylated with succinic anhydride at pH 10.5 and a temperature of 38 °C, as previously reported. Detailed protocol is provided in Sections S1.1 and S1.2 of the Supplementary Information. Hollow and curcumin-loaded protein and protein-chitosan complexes were prepared with the native (PPI) and succinylated pea protein isolates (SPPI), as briefly described in Scheme 1; detailed experimental procedures are presented in Section 3.2.1, Section 3.2.2, Section 3.2.3 and Section 3.2.4.

#### 3.2.1. Preparation of Reaction Solutions

Protein solutions of PPI and SPPI (5 mg/mL) were prepared in Milli-Q water, while solutions of Chitosan (2.5 mg/mL) and curcumin (1 mg/mL) were prepared in acetic acid (1%) and ethanol, respectively. The containers of curcumin solutions were wrapped with aluminum foil to protect curcumin from photodegradation. Protein and chitosan solutions were stirred for 6 h and centrifuged with a Sorvall LYNX 4000 superspeed centrifuge (ThermoFisher Scientific, Waltham, MA, USA) at 40,000× *g* for 30 min. The supernatant was vacuum filtered (Whatman filter paper number 50, pore size 2.7 μm) and degassed with Polylab vacuum desiccator to ensure air bubbles and gaseous impurities are removed.

#### 3.2.2. Preparation of Curcumin-Loaded Native and Succinylated Pea Protein Nano-Complexes

Curcumin-loaded native and succinylated pea protein nano-complexes were synthesized by anti-solvent precipitation, following a previously-reported protocol [1]. Briefly, each curcumin solution in ethanol (1 mg/mL, 0.5 mL) was added, dropwise, to a solution of PPI and SPPI (5 mg/mL, 2.0 mL) in a separate flask, while stirring at 320× *g* for 5 h, until a steady negative zeta-potential was maintained. The colloidal solutions were centrifuged at 7500× *g* for 30 min using an Eppendorf 5430R centrifuge (Eppendorf, Mississauga, ON, Canada). The precipitate obtained was washed twice with Milli-Q water and redissolved in water (5 mL) for the dynamic light scattering and microscopic studies, or in leakage buffer for the GUV leakage assay.

#### 3.2.3. Preparation of Curcumin-Loaded Protein-Chitosan Nano-Complexes

The synthesis of curcumin-loaded native/succinylated pea protein-chitosan nano-complexes was carried out by first synthesizing the curcumin-protein nano-complexes (0.5 mL curcumin, 1.5 mL protein) by anti-solvent precipitation, as described in Section 3.2.2. After stirring the colloidal solution at 320× *g* for 3 h, chitosan was incorporated through electrostatic deposition method, by dropwise addition of chitosan solution (2.5 mg/mL, 0.5 mL), while maintaining stirring for additional 5 h. The mixture was centrifuged at 7500× *g* for 30 min after obtaining a steady positive zeta-potential. The precipitate obtained was washed twice with water and redispersed, in water or buffer, for further studies.

#### 3.2.4. Preparation of Native/Succinylated Pea Protein-Chitosan Hollow Nano-Complexes

Hollow native and succinylated protein-chitosan nano-complexes were prepared by the electrostatic deposition method, as previously reported [1]. Briefly, each chitosan solution (2.5 mg/mL, 0.5 mL) was gradually added to a separate solution of native and succinylated pea proteins, while stirring at 320× *g* for 5 h. The solution was monitored for steady positive zeta-potential, after which it was centrifuged at 7500× *g* for 30 min. The precipitate was washed twice with water and redispersed, in water or buffer, for subsequent analysis. 

### 3.3. Interactions of the Protein Nanoparticles with Calcein-Loaded GUV

#### 3.3.1. Preparation of Empty and Calcein-Loaded Giant Unilamellar Vesicles

Zwitterionic giant unilamellar vesicles were prepared, according to a previously reported method [20], with some modifications. Briefly, a solution of 1,2-dioleoyl-sn-glycero-3-phosphocholine (7.2 mg/mL) was prepared in chloroform in a 50-mL centrifuge tube, covered with aluminum foil, and allowed to dry under a stream of nitrogen for 4 h, followed by vacuum drying for an additional 12 h. Calcein solution (20 mM, 15 mL) was prepared in the leakage buffer (10 mM Tris, 150 mM NaCl, 1 mM EDTA, pH 7.4) and pH adjusted to 7.5 with NaOH (0.2 M). The dried lipid film (36 mg) was rehydrated with this solution to reach a final lipid concentration of 2 mg/mL. The colloidal solution was vortexed for 1 min, sonicated at 30 °C for 1 h, and subjected to five freeze/thaw cycles to enhance the encapsulation of calcein in the vesicle. Each cycle was comprised of 3 min in liquid nitrogen, 5 min in 60 °C water bath, and vortex mixing for 1 min. Size exclusion column (sephadex G-50) was used to separate the calcein-loaded GUV from unencapsulated calcein, using a leakage buffer for equilibration and elution. The eluted calcein-loaded GUV was stored for a maximum of 72 h at 4 °C and used for the leakage study. 

Similarly, fluorescence-labeled calcein-loaded and free GUV was prepared for visualizing the impact of nanoparticle interaction. In this case, a mixture of 1,2-dioleoyl-sn-glycero-3-phosphocholine (20 mg) and 1,2-dioleoyl-sn-glycero-3 phosphoethanolamine-*N*-(lissamine rhodamine B sulfonyl) (ammonium salt) (1 mg) was dissolved in chloroform.

#### 3.3.2. Zwitterionic GUV Leakage Assay

The influence of nanoparticles on the structural integrity of zwitterionic GUV was investigated by fluorescence spectroscopy using Varian Cary Eclipse (Agilent, Santa Clara, CA, USA), as previously reported [20], with minor modifications. The eluted calcein-loaded GUV was diluted two-fold before mixing with equal volume of colloidal solutions of hollow, curcumin-loaded protein, and curcumin-loaded protein-chitosan nano-complexes, to reach final nanoparticle concentrations of 42 to 140 µg/mL. The spectrofluorometer was first zeroed with pure leakage buffer, and a fluorescence emission scan was performed, from 500 to 625 nm, at the excitation wavelength of 495 nm (calcein) and slit width of 2.5 nm. The fluorescence intensity of the calcein-loaded GUV was monitored for 10 min, to ensure stabilization of the signal, and used as the baseline fluorescence (F_0_). Fluorescence intensity (F) was measured after interaction of the calcein-loaded GUV with various concentrations of nanoparticles. The maximum fluorescence intensity, corresponding to the fluorescence intensity of calcein after 100% leakage (F_M_), was achieved with equal volume of 10% Triton X-100. The zwitterionic GUV leakage induced by the protein nanoparticles was estimated using to Equation (1).

(1) $$\%\mathrm{Leakage}\;=\frac{\left(F\;-{\;F}_{0}\right)}{\left(F_{M}-{\;F}_{0}\right)}\times 100$$

### 3.4. Morphological Characterization of the Structural Integrity of Zwitterionic GUV after Interaction with Nanoparticles

The effect of interactions of hollow or various curcumin-loaded nano complexes on the structural integrity of zwitterionic GUV was visualized with confocal and widefield fluorescence microscopes. Each solution of calcein-loaded GUV (500 µL) was mixed with a solution of the nanoparticles (150 µL, 0.28 mg/mL) or 10% Triton X-100 (150 µL) prepared in a leakage buffer, vortexed for 1 min, and allowed to stand for 15 min before mounting on glass microscope slides. To image calcein-loaded and empty GUV in the solution after leakage, the morphology was visualized separately in the rhodamine B (λ_ex_ 540, λ_em_ 625) and calcein (λ_ex_ 494, λ_em_ 517) channels, with the Axio Imager 2 fluorescence microscope equipped with an Axiocam 506 monochromatic camera (Carl Zeiss, Oberkochen, Germany) and Zeiss LSM880 AxioObserver Z1 confocal microscope equipped with AiryScan FAST (Carl Zeiss, Oberkochen, Germany). Zen 2.3 pro and 3.4 software (Carl Zeiss, Oberkochen, Germany) were used for image processing and optimization.

### 3.5. Dynamic Light Scattering Analysis

The impact of GUV-nanoparticle interaction on the particle size, polydispersity index, and zeta potential of calcein-loaded GUV was evaluated with the dynamic light scattering technique, using Nano-ZS Zetasizer (Malvern Instrument Ltd., Malvern, UK). Solutions of the calcein-loaded GUV (500 µL) and nanoparticles (150 µL, 0.28 mg/mL) were mixed and analyzed with the Smoluchowski model at absorption of 0.001, refractive index 1.450, F(ka) 1.50, and backscattered angle of 173° at 25 °C, after triplicate measurements.

### 3.6. Statistical Analysis

Experiments were conducted in triplicate, and mean values of data from leakage assay and dynamic light scattering were compared by one-way analysis of variance, using the Turkey’s test and group mean differences, in Origin 9 software (OriginLab Corporation, Northampton, MA, USA). Significant differences were defined at *p* < 0.05.

## 4. Conclusions

The bio-nano interaction of nutraceutical-loaded protein nanoparticles was investigated using hollow and curcumin-loaded protein nanoparticles of different surface chemistry (with or without chitosan or curcumin) and physicochemical properties (size, zeta potential, morphology, binding strength) as model delivery vehicles, with GUV as model biomembranes. Protein nanoparticle-lipid membrane interaction is mainly influenced by the composition, physicochemical property, and surface chemistry of nanoparticles. Interaction of CUR/PPI and CUR/SPPI nanoparticles, which have a smaller size range, higher negative zeta potential, spherical morphology, and lower binding strength, compared to CUR/SPPI/CHI and CUR/PPI/CHI, where calcein-loaded GUV led to the quenching of calcein fluorescence. Unlike CUR/PPI/CHI (effective binding constant 14.5 × 10^4^ M^−1^) that induced minimal calcein leakage, interaction of CUR/SPPI/CHI nanoparticles (effective binding constant 187.9 × 10^4^ M^−1^) with calcein-loaded GUV did not cause significant change in the morphology and structure of GUV or calcein fluorescence. Conversely, PPI/CHI and SPPI/CHI hollow with irregular morphology caused major rupturing of calcein-loaded GUV, leading to increased calcein leakage. Therefore, we propose two mechanisms for the nanoparticle-membrane interactions: (1) nanoparticle binding on membrane surface induces osmotic pressure, resulting in water escape from the membrane and consequent shrinkage; (2) nanoparticle binding on the membrane induces GUV bursting. The former was demonstrated by CUR/PPI and CUR/SPPI nanoparticles, while CUR/SPPI/CHI, CUR/PPI/CHI, PPI/CHI, and SPPI/CHI displayed the latter mechanism. CUR/SPPI/CHI nanoparticles with the highest stability and, hence, minimal impact to the biomembrane could be potentially less deleterious during delivery of bioactive compounds. Therefore, as protein and protein-chitosan complexes protect the bioactive compounds from photo and chemical degradation, the encapsulated nutraceutical could modulate the interaction of the protein-based nanoparticle with the gastrointestinal cell membrane. This study provides the first evidence of the implication of interactions at the bio-nano interface, for food protein-based nanodelivery systems. This information will guide the design of delivery systems that safely interact with biological membranes, while ensuring the protection of the guest compound from degradation and its sustained release at the target location in the body.

## Figures and Tables

**Table 1:** Changes in size, polydispersity index, and zeta potential of calcein-loaded GUV after interaction with the hollow or curcumin-loaded protein nanoparticles or Triton X-100.

Samples	Size (nm)	PDI	Zeta Potential (mV)
lGUV	1933.3 ± 29.0 ^b^	0.30 ± 0.03 ^a,b,c^	−5.7 ± 0.2 ^b^
lGUV + CUR/PPI	1091.3 ± 5.0 ^e^	0.33 ± 0.04 ^a,b,c^	−10.0 ± 0.1 ^e,f^
lGUV + CUR/SPPI	1028.7 ± 26.2 ^e^	0.25 ± 0.02 ^a,b,c^	−11.5 ± 0.3 ^f,g^
lGUV + CUR/PPI/CHI	1689.7 ± 55.2 ^c^	0.42 ± 0.10 ^a,b^	−9.6 ± 0.3 ^d,e^
lGUV + CUR/SPPI/CHI	2222.7 ± 36.4 ^a^	0.18 ± 0.05 ^b,c^	−7.9 ± 0.4 ^c,d^
lGUV + PPI/CHI	1464.7 ± 64.8 ^d^	0.41 ± 0.06 ^a,b^	−11.8 ± 0.2 ^g^
lGUV + SPPI/CHI	1551.7 ± 20.0 ^c,d^	0.43 ± 0.05 ^a^	−7.4 ± 0.2 ^b,c^
lGUV + Triton X-100	10.7 ± 0.0 ^f^	0.10 ± 0.00 ^c^	−1.0 ± 0.8 ^a^

Mean values with different letters in a column are significantly different (*p* < 0.05) Abbreviations: CUR/SPPI, curcumin-succinylated pea protein complex; CUR/PPI, curcumin-pea protein complex; CUR/SPPI/CHI, curcumin-succinylated pea protein-chitosan complex; CUR/PPI/CHI, curcumin-pea protein-chitosan complex; SPPI/CHI hollow, succinylated pea protein-chitosan shell; PPI/CHI hollow, native pea protein-chitosan shell; lGUV, giant unilamelar vesicles loaded with calcein.

## Data Availability

The article contains data supporting the findings.

## Supplementary Materials

The following supporting information can be downloaded at: https://www.mdpi.com/article/10.3390/molecules27061941/s1, Section S1.1: Pea protein extraction; Section S1.2: Succinylation of the pea protein isolate; Figure S1: Widefield fluorescence microscopy images of (**a**) empty GUV (**b**) GUV-loaded calcein in the absence of nanoparticles, and after interaction with (**c**)CUR/SPPI (**d**) CUR/PPI (**e**) CUR/SPPI/CHI (**f**) CUR/PPI/CHI (**g**) SPPI/CHI (**h**) PPI/CHI nanoparticles and (**i**) Triton-X-100 (10%) positive control. Images in the first column were acquired at calcein channel, second column at rhodamine B channel (GUV labeled with rhodamine), and third column is the merger of both.

## Sample Availability

Samples of the compounds are not available from the authors.
