# Supplementary material for: Curcumin–Induced Stabilization of Protein–Based Nano-Delivery Vehicles Reduces Disruption of Zwitterionic Giant Unilamellar Vesicles
Source: Molecules. 2022 Mar 17;27(6):1941. doi: 10.3390/molecules27061941 (PMC8956123; doi:10.3390/molecules27061941)
Supplement: Supplementary file 1 [file molecules-27-01941-s001.zip › molecules-1623247-supplementary.pdf]

Curcumin–Induced Stabilization Of Protein–Based Nano-Delivery Vehicles Reduces Disruption Of Zwitterionic Giant Unilamellar Vesicles

Ogadimma D. Okagu[a], Raliat O. Abioye[a] and Chibuike C. Udenigwe[*,a,b]

[a] Department of Chemistry and Biomolecular Sciences, Faculty of Science, University of Ottawa, Ottawa, ON, K1N 6N5, Canada
[b] School of Nutrition Sciences, Faculty of Health Sciences, University of Ottawa, Ottawa, ON, K1H 8M5, Canada
[*] Correspondence: University Research Chair in Food Properties and Nutrient Bioavailability, School of Nutrition Sciences, 451 Smyth Road, University of Ottawa, Ottawa, ON, Canada. Email: cudenigw@uottawa.ca; Tel.: +1 (613) 562-5800 ext. 6539

S1. Methodology

S1.1. Pea protein extraction

Isoelectric precipitation technique was employed in extracting pea protein isolate (PPI) from yellow pea seeds (*Pisum sativum*), as previously reported [1]. Briefly, dehulled yellow pea seeds were ground and sieved, and the flour (200 g) is extracted with NaOH solution (0.05 M, 2L) at pH 12.7, after 4 h of stirring in a magnetic stirrer (Thermo Scientific, Waltham, MA, USA). The resulting slurry was centrifuged at 25 °C and 5600× *g* for 30 min. The supernatant was separated from the residue, and pH adjusted to 4.5 with HCl (1 M), to precipitate PPI. The mixture was, again, centrifuged for 30 min and PPI collected, washed twice with Milli-Q water, and pH adjusted to 7. The PPI paste was frozen at −80 °C for 24h and lyophilized (Labconco, Kansas, MO, USA) at −52 °C and 0.2 mbar. The total protein content, as determined by the Lowry assay, was 96.1 %.

S1.2. Succinylation of the Pea Protein Isolate

Pea protein isolate was succinylated, according to a previously reported method [1]. It involves a drop-wise addition of a solution of succinic anhydride (0.5 M, 4 mL) to a preheated solution of PPI (100 mL, 2% *w*/*v*, 38 °C, pH 11), and the pH maintained at 10.5 with NaOH (1 M), while stirring for 4 h. The mixture was dialyzed against deionized water, with a membrane of molecular weight cut-off of 3.5 kDa for 48 h at 4 °C. The retentate was lyophilized and the powder was stored at −20 °C.

Supplementary material

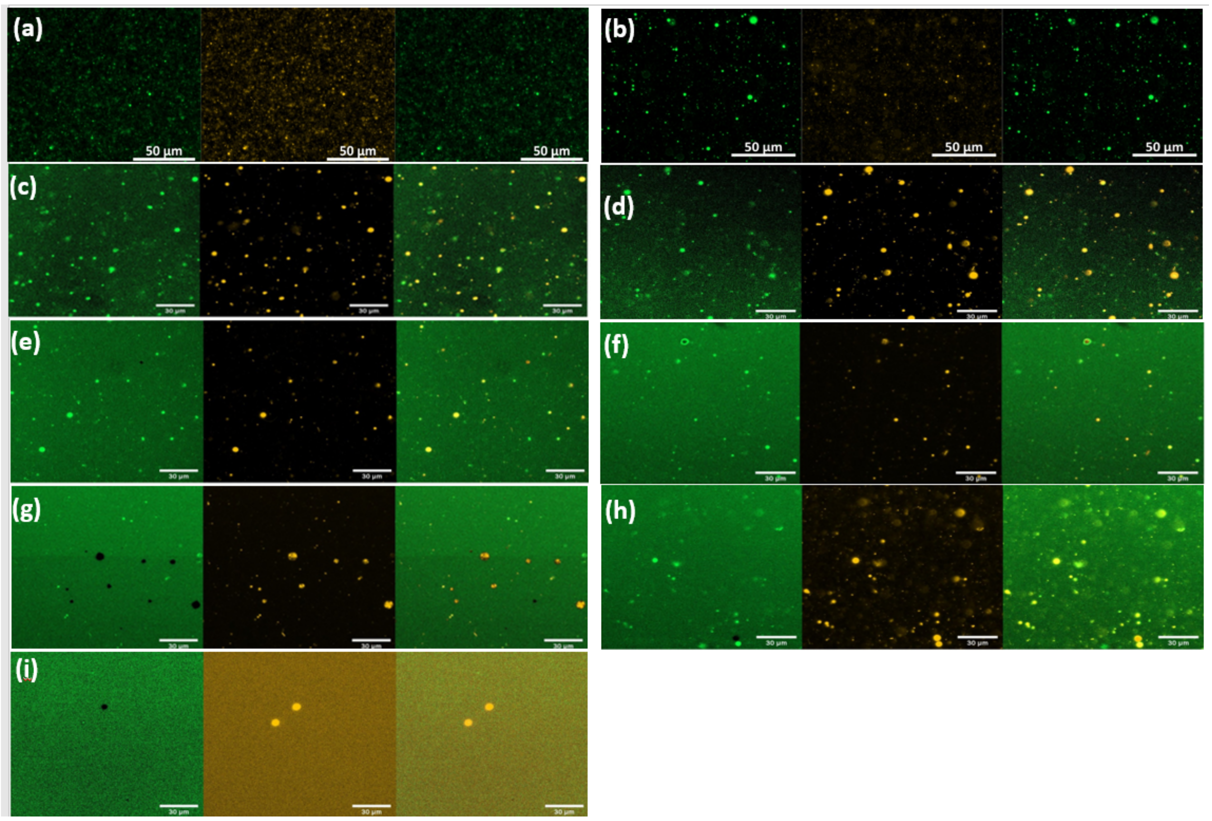

Figure S1. Widefield fluorescence microscopy images of (**a**) empty GUV, (**b**) GUV-loaded calcein in the absence of nanoparticles, and after interaction with (**c**)CUR/SPPI, (**d**) CUR/PPI, (**e**) CUR/SPPI/CHI, (**f**) CUR/PPI/CHI, (**g**) SPPI/CHI, (**h**) PPI/CHI nanoparticles, and (**i**) Triton-X-100 (10%) positive control. Images in the first column were acquired at calcein channel, second column is at rhodamine B channel (GUV labeled with rhodamine), and third column is the merger of both.
